# Genome-wide study of immune biomarkers in cerebrospinal fluid and serum from patients with bipolar disorder and controls

**DOI:** 10.1038/s41398-020-0737-6

**Published:** 2020-02-05

**Authors:** Ruyue Zhang, Jie Song, Anniella Isgren, Joel Jakobsson, Kaj Blennow, Carl M. Sellgren, Henrik Zetterberg, Sarah E. Bergen, Mikael Landén

**Affiliations:** 1 Department of Medical Epidemiology and Biostatistics, Karolinska Institutet, Stockholm, Sweden; 2 Department of Psychiatry and Neurochemistry, Institute of Neuroscience and Physiology, the Sahlgrenska Academy at the University of Gothenburg, Mölndal, Sweden; 3 Clinical Neurochemistry Laboratory, Sahlgrenska University Hospital, Mölndal, Sweden; 4 Department of Physiology and Pharmacology, Karolinska Institutet, Stockholm, Sweden; 5 Centre for Psychiatry Research, Karolinska Institutet, & Stockholm Health Care Services, Stockholm County Council, Karolinska University Hospital, Stockholm, Sweden; 6 Department of Neurodegenerative Disease, UCL Queen Square Institute of Neurology, Queen Square, London, UK; 7 UK Dementia Research Institute at UCL, London, UK

**Keywords:** Genomics, Clinical genetics

## Abstract

Bipolar disorder is a common, chronic psychiatric disorder. Despite high heritability, there is a paucity of identified genetic risk factors. Immune biomarkers are under more direct genetic influence than bipolar disorder. To explore the genetic associations with immune biomarker levels in cerebrospinal fluid (CSF) and blood serum which previously showed differences in bipolar disorder, we performed a study involving 291 individuals (184 bipolar disorder patients and 107 controls). The biomarkers assayed in both CSF and serum were: chitinase-3-like protein-1 (YKL-40), monocyte chemoattractant protein-1 (MCP-1), soluble cluster of differentiation (sCD14), tissue inhibitor of metalloproteinases-1 and 2 (TIMP-1 and TIMP-2). C-reactive protein (CRP) was only quantified in serum, and interleukin 8 (IL-8) measures were only available in CSF. Genome-wide association studies were conducted using PLINK for each of three genotyping waves and incorporated covariates for population substructure, age, sex, and body mass index (BMI). Results were combined by meta-analysis. Genome-wide significant associations were detected for all biomarkers except TIMP-1 and TIMP-2 in CSF. The strongest association in CSF was found for markers within the *CNTNAP5* gene with YKL-40 (rs150248456, *P* = 2.84 × 10^−10^). The strongest association in serum was also for YKL-40 but localized to the *FANCI* gene (rs188263039, *P* = 5.80 × 10^−26^). This study revealed numerous biologically plausible genetic associations with immune biomarkers in CSF and blood serum. Importantly, the genetic variants regulating immune biomarker levels in CSF and blood serum differ. These results extend our knowledge of how biomarkers showing alterations in bipolar disorder are genetically regulated.

## Introduction

Bipolar disorder is a chronic psychiatric disorder characterized by recurrent episodes of mania or hypomania and depression that afflicts about 60 million people worldwide^1–3^. The etiopathogenesis of bipolar disorder is not fully understood. Despite heritability estimates ranging between 58–93%^4–6^, only a few dozen single nucleotide polymorphisms (SNPs) with small effect sizes have been associated with bipolar disorder in large-scale genome-wide association studies (GWAS)^7–11^.

One line of research has explored immuno-inflammatory processes in bipolar disorder and a number of studies have investigated immune markers in serum^12–16^. Serum immune markers are, however, not necessarily indicative of immune and inflammatory activity in the brain. This is because concentrations of cytokines and other proteins in serum or plasma come from production in peripheral tissues and thus do not reflect inflammatory processes in the brain^17–19^. Therefore, we previously investigated a set of immune biomarkers in cerebrospinal fluid (CSF) in bipolar disorder and healthy controls, including monocyte chemoattractant protein-1 (MCP-1), chitinase-3-like protein-1 (YKL-40), soluble cluster of differentiation 14 (sCD14), tissue inhibitor of metalloproteinases-1/2 (TIMP-1 and TIMP-2), and interleukin 8 (IL-8)^20,21^. Taken together, these results suggest that brain-specific immune mechanisms, beyond systemic inflammatory processes, are involved in the pathophysiology of bipolar disorder.

Immune biomarkers are products of gene expression and are as such measurable components in biological pathways between genotype and disease. Immune biomarkers are therefore potential endophenotypes for some psychiatric disorders by representing a measurable characteristic of the disorder more closely related to the genetic underpinnings than behavioral manifestations such as bipolar disorder^15,22,23^. Available evidence indicates a number of loci associated with immune biomarker levels among different populations^15,24–26^, but few genome-wide association studies of multiple immune biomarkers have been published in bipolar disorder cohorts. Revealing genetic associations for CSF and blood serum biomarkers serving as indicators of biological processes implicated in bipolar disorder might yield new insights into the genetic underpinnings of bipolar disorder.

The aim of this study was to conduct a GWAS of a set of immune biomarkers in CSF and serum that previously have been found to differ between bipolar disorder patients and controls.

## Materials and methods

### Subjects

The study population consisted of subjects with bipolar disorder as well as age- and sex- matched controls. Subjects were recruited from a long-term naturalistic study of bipolar disorder, the St. Göran Bipolar Project, at the bipolar outpatient unit at the Northern Stockholm psychiatric clinic in Stockholm, Sweden. The work-up and diagnostic procedures for patients and selection of controls have been described in detail previously^20,27–29^. In brief, the key clinical assessment instrument was a Swedish version of the Affective Disorder Evaluation (ADE), which is a standardized interview protocol developed for the Systematic Treatment Enhancement Program of Bipolar Disorder (STEP-BD). The clinical diagnosis of bipolar disorder was made according to DSM-IV criteria as per the Structured Clinical Interview for DSM-IV. In addition, the Mini International Neuropsychiatric Interview (M.I.N.I.) was completed to screen for other psychiatric diagnoses. The ADE and M.I.N.I. interviews were conducted by board-certified psychiatrists, or residents in psychiatry. A best-estimate diagnostic decision was then made based on all information available by a consensus panel of experienced board-certified psychiatrists specialized in bipolar disorder. To gather a representative cohort of bipolar disorder patients, the St. Göran study aimed to have as few exclusion criteria as possible and persons with somatic disorders including autoimmune disorder were not excluded. Patients were not remunerated for participation.

Population-based controls living in the same catchment areas were randomly selected by Statistics Sweden and contacted by mail. Details of the recruitment, and inclusion and exclusion criteria can be found elsewhere^29,30^. Briefly, eligible persons were scheduled for a personal examination and investigated to exclude mental illness by a psychiatrist using the Mini International Neuropsychiatric Interview (M.I.N.I.) and selected parts of the ADE. Exclusion criteria were as follows: any current psychiatric disorder including personality disorder, a family history of schizophrenia or bipolar disorder in first-degree relatives, drug or alcohol abuse (based on DUDIT, AUDIT and serum levels of carbohydrate-deficient transferrin), and neurological conditions except mild migraines, as well as pregnancy, untreated endocrine disorders, dementia, and chronic systemic autoimmune disorders, except persons with controlled asthma and allergies. Control subjects were remunerated for their participation.

Only cases and controls with Scandinavian ancestry were included in this study. All participating subjects granted oral and written informed consent after complete description of the study. Ethical approvals for this study were granted by the Stockholm Regional Ethics Committee.

### Sampling and biomarker analyses of CSF and blood

CSF and blood were obtained when the participants were in a stable condition. Sampling occurred between 9 and 10 am after an overnight fast. For each participant, 12 mL of CSF was collected and gently inverted to avoid gradient effects. Serum was obtained from blood samples after coagulation and centrifugation. Both serum and CSF samples were stored at −80 °C at Karolinska Institutet Biobank, Sweden. The assays and kits for each biomarker have been described in detail previously^20,21^. All biomarker concentrations were measured by experienced and board-certified laboratory technicians at the Clinical Neurochemistry Laboratory in Mölndal, Sweden who were blinded to the clinical information.

### Genotyping, quality control, and imputation

Blood samples were transferred to the Karolinska Institutet Biobank for DNA extraction. An aliquot of each DNA sample was shipped to the Broad Institute (Boston, USA) for genotyping. Whole-genome genotyping was conducted at the Broad Institute using PsychChip (wave 1), Affymetrix 6.0 (wave 2), and Illumina OmniExpress (wave 3) chips. All controls were in wave 1 and wave 3, while the bipolar cases were exclusively in wave 2. Following quality control steps, datasets were then imputed with the full 1000 Genomes Project integrated variant set as reference^31^. Details of genotyping, quality control, and imputation procedures have been described previously^32^. The three waves shared more than 10 million SNPs after imputation.

A total of 183 bipolar cases and 107 controls from the St. Göran project had both biomarker information and genotype data available.

### Statistical analyses

We used PLINK version 1.9, SPSS Statistics 23 and R version 3.3.3 with the qqman package for all statistical analyses. Group differences between bipolar cases and controls were tested using the Mann-Whitney U test for age, BMI, and all biomarker concentrations, while Fisher’s exact test was used for sex. Interquartile range was calculated for continuous variables.

The CRP distributions deviated from normality, prohibiting use of linear regressions in GWAS analysis. We therefore conducted a log transformation to adapt it to a distribution compatible with linear regression.

The GWAS analysis was conducted using imputed SNP dosages and linear regression models in PLINK for each wave and incorporated the first four multidimensional scaling (MDS) components, age, sex, and BMI as covariates. Results were then combined by meta-analysis in PLINK using a random-effects model. Although imputed data were used, there were still SNPs that were not present in one or more waves. Waves included in the meta-analysis are noted in results. SNPs with minor allele frequency (<1%) and poor imputation (INFO < 0.6) were removed from meta-analysis results. A standard genome-wide significance threshold of *p* < 5 × 10^−8^ was used^33^. Linkage disequilibrium based clumping was used for grouping correlated SNPs to define regions of association. Genes were also identified based on UCSC hg19 coordinates. GWAS summary statistics were also uploaded to FUMA to obtain results from MAGMA gene-set analysis and MAGMA tissue expression analysis (GTEx v6, 30 general tissue types)^34,35^.

SNPs with p-values < 5 × 10^−5^ from either CSF or serum results were used for the difference test to compare the effect sizes between CSF and serum results. Effect sizes of these SNPs were standardized by dividing by the standard deviations of the biomarker levels. Pearson correlation coefficients were used to test the correlation of standardized effect sizes from CSF and serum results.

### Code availability

The scripts used to generate these results can be provided upon request.

## Results

### Demographics and clinical characteristics

We included 184 bipolar disorder patients (67 men and 117 women) and 107 controls (46 men and 61 women) for the serum measurements. A subset of this sample population—114 bipolar disorder patients (44 men and 70 women) and 83 controls (36 men and 47 women)—who consented to lumbar puncture comprised the study population for CSF measurements. A summary of sample sizes included in this study for each genotyping wave is described in Supplementary Table S1.

A description of demographic and clinical characteristics of the study population as well as comparison of differences between bipolar patients and controls can be found in Supplementary Table S2. Note that the comparisons of biomarker concentrations between cases and controls shown in Table S2 have been published previously^20,21^, but may differ somewhat as not all individuals had both biomarker information and genotype data available.

### Genetic variants associated with immune biomarkers in CSF

A total of >5.6 million SNPs from 114 bipolar patients and 83 controls were included in our final meta-analysis for biomarkers in CSF. The quantile-quantile plots for each of the six biomarkers showed moderate deviations from the null distribution at low *p*-values indicating the presence of association signals, but no deviation at the higher *p*-values, which denotes well matched cases and controls, i.e., no inflation (Fig. S1). The genomic inflation factor, λ, ranged from 1.05–1.11.

**Fig. 1:**
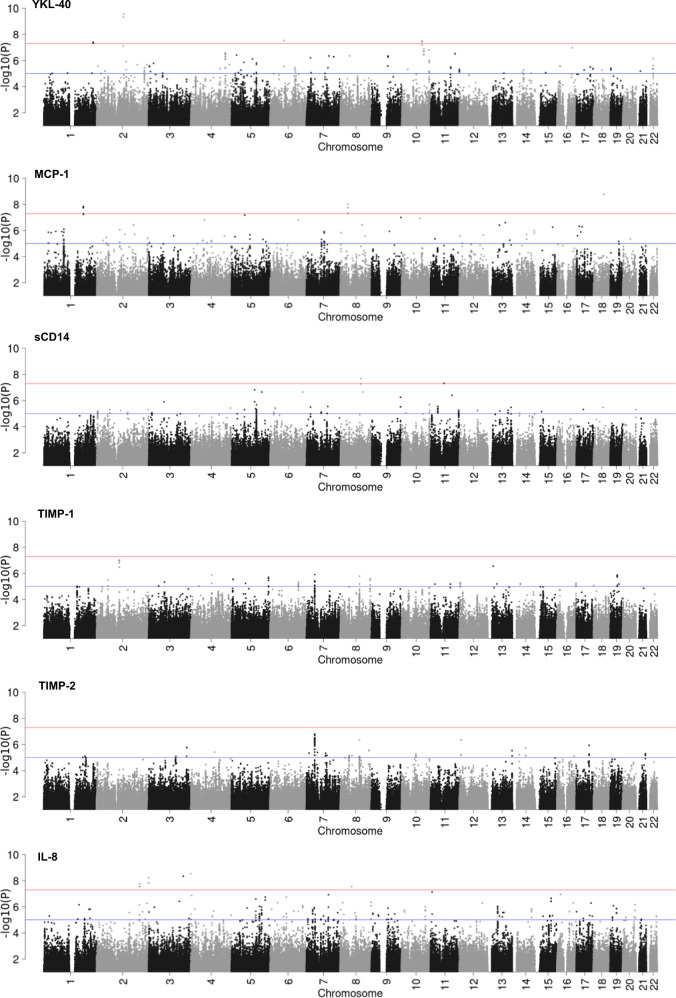
Manhattan plots for six CSF immune biomarkers. For each of the six biomarkers, a Manhattan plot shows the −log10 *P*-value (vertical axis) for all SNPs that pass the standard quality control filters, have a minor allele frequency > 1% and imputation info score > 0.6 along with the chromosome position (horizontal axis). Chromosomes are shown in alternating colors for clarity. The red lines denote genome-wide significant *P*-values < 5 × 10^−8^ while the blue lines show suggestive associations with *P*-values < 1 × 10^−5^.

The genome-wide association analysis results for the six biomarkers in CSF are shown in Fig. 1. The number of genome-wide significant regions (Index SNPs’ *P*-values < 5 × 10^−8^) for each GWAS meta-analysis on YKL-40, MCP-1, sCD14, and IL-8 in CSF was 4, 3, 2, and 6, respectively. No SNPs of genome-wide significance were associated with TIMP-1 and TIMP-2.

Table 1 illustrates the genome-wide significant (GWS) regions for each biomarker in CSF. The top GWS SNPs associated with YKL-40 were located in the genes *CNTNAP5, EYS*, and *FER1L3*. For MCP-1, the SNP with the strongest association was located within *ACAA2* gene (rs10438979, P_A/G_ = 1.64 × 10^−9^). The other two top GWS SNPs were located near or within *LINCO1288*, *FAM129A*, and *EDEM3*. Two GWS SNPs were related to sCD14 levels in CSF, which are located in the gene area of *C8orf37-AS1* and *TUT1*.

**Table 1:** Top GWS regions for each biomarker in CSF.

Chr	Index SNP	A1/A2	Freq	ß	*p*-value	N	Position	KB	Genes	Waves
*YKL-40*										
2	rs150248456	A/G	0.989	−88.276	2.84e-10	2	chr2:125287495..125383005	95.51	***CNTNAP5***	1,3
6	rs11753319^a^	C/G	0.951	−39.387	2.89e-08	2	chr6:64902720..65013122	110.40	***EYS***	1,2,3
10	rs182911102^a^	T/C	0.985	−64.832	3.15e-08	4	chr10:94991992..95075604	83.61	***FER1L3***	1,2,3
1	rs16856846	A/T	0.020	62.660	4.11e-08	3	chr1:232470911..232485096	14.19		1,2
*MCP-1*										
18	rs10438979^a^	A/G	0.960	−230.358	1.64e-09	1	47267946	0	*ACAA2*	1,2,3
8	rs193114076^a^	A/G	0.011	370.998	9.68e-09	3	chr8:34726827..34789467	62.64	*LINC01288*	1,2,3
1	rs114271760	A/G	0.014	362.410	1.45e-08	5	chr1:184697347..184966318	268.97	*FAM129A,EDEM3*	1,3
*sCD14*										
8	rs182557857	T/C	0.012	94.392	2.05e-08	4	chr8:96576432..96626315	49.88	***C8orf37-AS1***	1,3
11	rs117456286	A/G	0.013	89.513	4.76e-08	1	62345761	0	***TUT1***	1,3
*IL-8*										
4	rs142700748^a^	T/C	0.975	−20.461	2.82e-09	1	234362	0	***ZNF876P***	2,3
3	rs184700722	T/C	0.011	−16.515	4.41e-09	1	162966687	0	***LINC01192***	1,3
2	rs182193570^a^	A/G	0.017	26.143	5.79e-09	1	242292698	0	***SEPT2***	2,3
2	rs187443429^a^	T/C	0.024	23.725	1.45e-08	1	242392862	0	***FARP2***	2,3
2	rs9646754^a^	T/C	0.034	20.001	1.74e-08	1	200300101	0	***SATB2***	1,2,3
2	rs115307848^a^	A/G	0.021	22.536	2.79e-08	3	chr2:199894134..200004958	110.82		1,2,3

Genes in bold include the index SNP. Genes not in bold are the gene area that SNPs are closest to. GWS is defined as *P* < 5e-08. A1/A2, beta (ß), *p*-value, waves are based on the meta-analysis of GWAS data from each wave. Position is the basepair position or given in UCSC hg19 coordinates.

*Chr* chromosome, (Index)*SNP* single nucleotide polymorphism (with the strongest association in the genomic region), *A1/A2* reference and alternate allele, *Freq* weighted average frequency of reference allele, *ß* random-effects meta-analysis ß estimate, *p-*value random-effects meta-analysis *p*-value, *N* number of SNPs in the reported region, *Waves* valid waves included for the SNP.

^a^Results from meta-analysis including wave 2.

The GWS SNPs associated with IL-8 were located in the genes *ZNF876P, LINC01192, SEPT2, FARP2*, and *SATB2*.

The results from MAGMA tissue expression analysis for 30 general tissue types for biomarkers from CSF are shown in Fig. S2. The genetic variants associated with YKL-40 from CSF were significantly expressed in bladder. No other significant findings from GTEx were identified.

### Genetic variants associated with serum immune biomarkers

Over five million SNPs from 184 bipolar patients and 107 controls were included in our final meta-analysis for biomarkers in serum. The quantile-quantile plots for each of the six biomarkers were consistent with no inflation and strong association signals (Fig. S3). The genomic inflation factor, λ, ranged from 1.01 to 1.05.

**Fig. 2:**
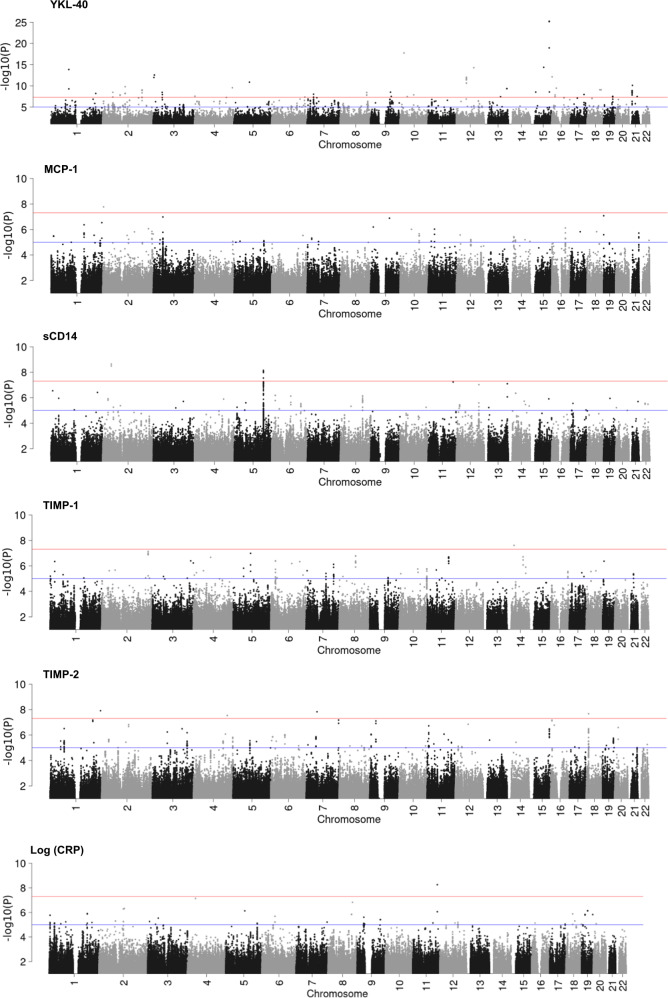
Manhattan plots for six serum immune biomarkers. For each of the six biomarkers, a Manhattan plot shows the −log10 *P*-value (vertical axis) for SNPs that pass the standard quality control filters, have a minor allele frequency > 1% and imputation info score > 0.6 along with the chromosome position (horizontal axis). Chromosomes are shown in alternating colors for clarity. The red lines denote genome-wide significant *P*-values < 5 × 10^−8^ while the blue lines show suggestive associations with *P*-values < 1 × 10^−5^.

The genome-wide association analysis results for the six biomarkers in serum are illustrated in Fig. 2. The serum biomarkers (and number of GWS regions) were: YKL-40 (*n* *=* 37), MCP-1 (*n* *=* 1), sCD14 (*n* *=* 2), TIMP-1 (*n* *=* 1), TIMP-2 (*n* *=* 4), and CRP (*n* *=* 1).

Table 2 shows the GWS regions for each biomarker in blood serum. Three top GWS SNPs associated with YKL-40 levels map to the gene *FANCI*. One SNP reached GWS for MCP-1 (rs34625674, P_D/I8_ = 1.70 × 10^−8^) and one for TIMP-1 (rs118161330, P_A/G_ = 2.50 × 10^−8^). The top GWS SNPs associated with sCD14 were located in the gene *SLC8A1*. Notably, after meta-analysis with all three waves, the largest number of SNPs (*n* = 110) associated with sCD14 were identified on chromosome 5q31.3 (top: rs2569191, P_T/C_ = 7.09 × 10^−9^, ß = −125.703, MAF = 0.421). More than 15 genes are located in this area. One gene-set remained significantly associated with sCD14 after Bonferroni correction (Table S3). As for TIMP-2, the largest number of SNPs (*n* = 36) were identified near the gene area of *LOC101927410* (top: rs150356358, P_A/G_ = 2.16 × 10^−8^, ß = −13.907, MAF = 0.211). The lone GWS SNP associated with log (CRP) was located within the *UBASH3B* gene.

**Table 2:** Top GWS SNPs for each immune biomarker in blood serum.

Chr	Index SNP	A1/A2	Freq	ß	*p*-value	N	Position	KB	Genes	Waves
*YKL-40*										
15	rs188263039	T/G	0.988	−76.738	5.80e-26	1	89809787	0	***FANCI***	1,3
15	rs117809422	A/G	0.015	76.377	7.24e-26	1	89815928	0	***FANCI***	1,3
15	rs188631178	T/C	0.986	−67.032	1.15e-19	1	89845859	0	***FANCI***	1,3
10	rs17602253^a^	T/C	0.988	−65.955	1.79e-18	1	18334915	0	*SLC39A12*	1,2
15	rs62012778^a^	A/C	0.966	−37.325	4.41e-15	4	chr15:63818629..63895362	76.73	***USP3,FBXL22***	1,2,3
1	rs116057745^a^	A/G	0.982	−31.092	1.47e-14	1	85746632	0		1,2,3
16	rs117868506^a^	T/C	0.013	50.578	7.44e-13	2	chr16:1420638..1428236	7.60	***UNKL***	2,3
12	rs117843845^a^	T/C	0.020	47.572	9.12e-13	8	chr12:48287373..48314900	27.527	VDR	1,2
3	rs182265848^a^	T/G	0.014	58.516	1.05e-12	1	chr3:2579507..2579507	0	CNTN4	2,3
5	rs35017964^a^	T/C	0.989	−48.149	1.33e-11	1	chr5:73222206..73222206	0		2,3
2	rs190160997^a^	A/C	0.011	45.776	1.58e-10	4	chr2:107295383..107436962	141.579	ST6GAL2	2,3
4	rs116622969^a^	T/C	0.983	−36.776	2.80e-10	1	chr4:182066384..182066384	0		1,2,3
16	rs112260868	A/G	0.012	53.408	3.50e-10	1	chr16:21613268..21613268	0	METTL9	1,3
13	rs150949191^a^	A/C	0.015	37.114	4.56e-10	2	chr13:109235412..109238352	2.94		2,3
1	rs114751860	T/C	0.013	40.152	5.25e-10	1	chr1:85883897..85883897	0	DDAH1	1,3
18	rs172586^a^	A/G	0.020	46.598	8.46e-10	1	chr18:60321603..60321603	0		2,3
18	rs512482^a^	T/G	0.978	−24.412	8.81e-10	1	chr18:66119026..66119026	0		2,3
2	rs186493621^a^	A/C	0.985	−36.147	9.29e-10	16	chr2:187931545..188399880	468.34	TFPI,CALCRL	2,3
21	rs28707003^a^	T/C	0.958	−28.903	1.33e-09	12	chr21:15202015..15222754	20.74		1,2,3
15	rs67659323^a^	C/G	0.960	−29,117	2.98e-09	1	chr15:25490757..25490757	0		1,2,3
9	rs117574354	C/G	0.015	25.396	3.08e-09	5	chr9:94685768..94923946	238.178	SPTLC1,ROR2	1,3
2	rs150886752^a^	T/C	0.017	40.299	3.36e-09	2	chr2:47882212..47901294	19.08		1,2,3
3	rs34307719^a^	T/C	0.987	−45,440	3.38e-09	1	chr3:42943216..42943216	0		1,2
8	rs960667^a^	T/G	0.976	−24,890	3.69e-09	5	chr8:126632214..126636054	3.84		1,2
2	rs116200463^a^	T/C	0.013	40.224	6.12e-09	3	chr2:107460348..107488170	27.82	ST6GAL2	2,3
1	rs2290199^a^	A/G	0.035	20.132	6.21e-09	3	chr1:214825751..214853690	27.94	CENPF	1,2,3
7	rs6969115^a^	A/G	0.018	35.247	9.34e-09	18	chr7:30250241..30281728	31.49		1,2
17	rs4791291^a^	T/C	0.924	−16,959	1.10e-08	4	chr17:65369397..65396158	26.76	PITPNC1	1,2,3
3	rs192944990^a^	T/C	0.012	40.733	1.14e-08	2	chr3:43867670..43888114	20.44		1,2
16	rs138053699^a^	A/G	0.019	46.309	1.40e-08	2	chr16:15812581..15821114	8.53	NDE1,MYH11	1,2
2	rs58011745^a^	A/G	0.035	25.632	1.68e-08	1	chr2:82922195..82922195	0		1,2,3
4	rs116608013^a^	A/C	0.987	−49,182	2.76e-08	1	chr4:2397776..2397776	0	ZFYVE28	1,2
9	rs112153762^a^	C/G	0.977	−39,896	3.10e-08	4	chr9:98468727..98480513	11.79		2,3
19	rs118145918^a^	T/G	0.024	32.162	3.13e-08	17	chr19:44419463..44487889	68.43	ZNF45,ZNF221	1,2
10	rs113755171^a^	T/C	0.011	45.313	3.47e-08	1	chr10:34100343..34100343	0		2,3
6	rs115196122^a^	A/G	0.983	−41.511	4.36e-08	3	chr6:159346245..159354145	7.9		1,2
7	rs11979716^a^	T/G	0.022	32.630	4.57e-08	8	chr7:30292334..30320400	28.07		1,2
*MCP-1*										
2	chr2_3403609_D^a^	D/I8	0.033	254.836	1.70e-08	1	3403609	0	TRAPPC12	1,2,3
*sCD14*										
2	rs190197089^a^	T/C	0.015	700.006	2.27e-09	2	chr2:40635776..40659665	0	***SLC8A1***	1,2
5	rs2569191^a^	T/C	0.579	−125.703	7.09e-09	110	139779258..140186438	407.18	***ZMAT2,WDR55,TMCO6,SRA1,SLC35A4,PCDHA2,PCDHA3,PCDHA1,NDUFA2,IK,HARS,HARS2,EIF4EBP3,DND1,CD14,APBB3,ANKHD1,ANKHD1-EIF4EBP3***	1,2,3
*TIMP-1*										
14	rs118161330	A/G	0.017	−140.235	2.50e-08	1	33801149	0	***NPAS3***	1,3
*TIMP-2*										
1	chr1_243283251_D^a^	D/I6	0.018	54.173	1.24e-08	1	243283251	0		1,2,3
7	rs117145170^a^	A/T	0.019	60.350	1.51e-08	1	52273737	0		1,2
18	rs150356358^a^	A/G	0.789	−13.907	2.16e-08	36	10640992..10656164	15	*LOC101927410*	1,2,3
4	rs114147769^a^	A/T	0.968	−42.516	2.94e-08	1	162690147	0	***FSTL5***	2,3
*log(CRP)*										
11	rs57213254	T/C	0.011	3.16	5.51e-09	2	chr11:122678513..122679916	1.40	***UBASH3B***	1,3

Bold gene names include the index SNP within the gene. Genes not in bold are those that SNPs are closest to. GWS is defined as *P* < 5e-08. A1/A2, beta(ß), p-value, waves are based on the meta-analysis of GWAS data from each wave. Position is the basepair position or given in UCSC hg19 coordinates.

*Chr* chromosome, *(Index)SNP* single nucleotide polymorphism (with the strongest association in the genomic region), *A1/A2* reference and alternate allele, *Freq* weighted average frequency of reference allele, *ß* random-effects meta-analysis ß estimate, *p-*value random-effects meta-analysis p-value, *N* number of SNPs in the reported region, *Waves* valid waves included for the SNP.

^a^Results from meta-analysis including wave 2.

The results from MAGMA tissue expression analysis for 30 general tissue types for biomarkers from serum are shown in Fig. S4. None demonstrated significance.

### Difference tests for top SNPs in CSF and serum

The genome-wide significant SNPs for serum and CSF for each biomarker with both measures did not overlap. To formally test for similarity in sub-significance threshold results, we compared the difference in effect sizes for top SNPs in both measures using a correlation test. The correlation coefficients of each biomarker pair and corresponding *p*-values are presented in Table 3.

**Table 3:** Pearson correlation coefficients (r) between standardized effect sizes of top SNPs from CSF and serum results.

Biomarkers	YKL-40	MCP-1	sCD14	TIMP-1	TIMP-2
r	0.533	0.449	0.092	−0.036	−0.008
*P*-value	<0.001*	<0.001*	0.020*	0.360	0.796
N	937	444	638	658	963

*N* number of SNPs included in the test.

*P-value < 0.05.

From this test, there were significant positive correlations between standardized effect sizes of top SNPs in different tissues in YKL-40 (r = 0.533, *P* < 0.001), MCP-1(r = 0.449, *P* < 0.001), and sCD14 (r = 0.092, *P* = 0.020). However, the correlation in sCD14 was rather modest while the standardized effect sizes of top SNPs from different tissues in YKL-40 and MCP-1 showed moderate correlation. The correlation coefficients of TIMP-1 (r = −0.036, *P* = 0.360) and TIMP-2 (r = −0.008, *P* = 0.796) were close to zero, which indicates no relationship between effect sizes of SNPs associated with CSF or serum.

## Discussion

This is the first genome-wide study of multiple immune biomarkers in serum and CSF in bipolar disorder. Despite the modest sample size, a number of SNPs reached genome-wide statistical significance.

### CSF markers

Among immune biomarkers in CSF, the strongest association was found between rs150248456 and YKL-40. Rs150248456 is located within an intron of *CNTNAP5* (contactin associated protein like 5) on chromosome 2. The product of *CNTNAP5* belongs to the neurexin family^36^. Interestingly, SNPs located in *CNTNAP5* have been found to be significantly associated with mathematical ability, self-reported educational attainment, cognitive performance and response to antipsychotic treatment in schizophrenia^37,38^. Two GWS SNPs—only represented in genotyping waves containing control subjects—were observed in this area. Although CSF concentration of YKL-40 differs between bipolar cases and controls^20^, genes are likely to regulate the expression of biomarkers in people with and without bipolar disorder in a similar way. Interestingly, a previous GWAS of bipolar disorder in Norwegian individuals followed by replication in Icelandic samples also found nominally significant (*P* < 0.05) markers located in this gene area^39^. The top significant region associated with CSF YKL-40 in bipolar disorder cases was in the gene *EYS* (rs11753319), which is a novel locus. SNPs located in *EYS* were also reported to be significantly associated with mathematical ability, self-reported educational attainment, BMI, alcohol drinking, systolic blood pressure, mood disorder, unipolar depression, and schizophrenia^37,40–43^. Several SNPs located near *LINC01288* and *EDEM3* were found to be genome-wide significantly associated with MCP-1. *LINC01288* was reported to be significantly associated with BMI, while *EDEM3* was found to be associated with educational attainment, mathematical ability, systemic lupus erythematosus, and cognitive performance^37,44,45^. Four GWS SNPs located in *C8orf37-AS1* were found to be associated with CSF concentration of sCD14. Heel bone mineral density and smoking behaviors have previously been associated with *C8orf37-AS1*.

Although no SNPs reached genome-wide significance for TIMP-1 or TIMP-2 concentration in CSF, several association peaks for the two biomarkers nearly reached GWS (Fig. 2). This suggests that larger sample sizes may yield significant associations.

Among genes associated with IL-8 in CSF, *SATB2* is highly expressed in brain^46^, indicating a potential molecular mechanism from associated SNPs to IL-8 CSF levels.

### Serum markers

As for immune biomarkers in serum, the top three GWS SNPs associated with YKL-40 are located in the intron area of gene *FANCI* on chromosome 15. The product of the *FANCI* gene is a member of the Fanconi anaemia complementation (FANC) group^46^. Genetic variation in *FANC* group has previously been found to be associated with psychiatric illness^47^. When considering both cases and controls, the top SNP is located near the gene *SLC39A12*. This gene is highly expressed in brain and was identified by bioinformatic analyses as a significant molecular biomarker in the progression of psychiatric disorders, including bipolar disorder^48^. Moreover, the genes *SPTLC1*and *ROR2* that were associated with YKL-40 level in serum are moderately associated with schizophrenia and bipolar disorder (*P* = 5.79 × 10^−7^) according to a previous GWAS^49^.

The strongest association with sCD14 in serum was with rs190197089. This SNP is located within an intron of *SLC8A1* (solute carrier family 8 member A1) on chromosome 2. There is another GWS SNP located in the same area. Both of them are from the meta-analysis of two waves (wave 1 and wave 2), but were unavailable in wave 3. Interestingly, the *SLC8A1* gene has previously been associated with bipolar disorder^50,51^. The largest number of SNPs (*n* = 110) associated with sCD14 after meta-analysis with all 3 waves were identified on chromosome 5q31.3 (top: rs2569191). More than 15 genes are located in this area. The 5q31.3 region has previously been linked to bipolar disorder^50^. The other top SNP associated with sCD14 in serum, rs2569191, is located near the *CD14* gene. *CD14* encodes a surface antigen that is expressed by monocytes^46^.

There was one GWS locus associated with TIMP-1 level in serum. It is located near *NPAS3*, which is a transcription factor involved in neurogenesis. *NPAS3* has been implicated in a pathway associated with bipolar disorder^52^. However, this was the result only from controls. Among genes where SNPs associated with TIMP-2 were located, *FSTL5* is the gene with the highest expression in brain among all tissues^53^.

After log transformation of CRP levels in serum, one SNP (rs57213254) was GWS. Altered CRP levels in serum have been found to be associated with not only bipolar disorder, but also hypertension, stroke, coronary heart disease, type 2 diabetes mellitus, and cancer^54–58^. A number of loci were associated with serum CRP levels in a previous GWAS in the general population^25^. However, the GWS SNP associated with CRP in this study is novel. Replication is needed to confirm the association between this GWS SNP and altered CRP levels in bipolar patients and controls.

Compared with previous studies in larger samples, we still found a number of novel GWS SNPs associated with disease-associated immune biomarker level differences. This might imply possible biological pathways from genetic markers to inflammatory mediators in bipolar disorder. However, additional work is needed to confirm this conjecture. There are several potential explanations for the new associations despite the small sample size: First, previous GWAS of biomarkers have studied the general population or people with Alzheimer’s disease^24,25^. The novel GWS SNPs found in the meta-analysis including wave 2 (which was case-only) might be associated with biomarker differences in bipolar disorder specifically. Second, although imputation was conducted on each wave, some SNPs did not exist across all three waves due to array differences or fluctuations in allele frequencies across waves. The GWS results from only the control waves which were not significant in prior studies^25,59^ have very low minor allele frequencies (MAF < 0.015). They might therefore have been removed from wave 2 during quality control steps and may not have been analyzed in other studies. However, the biomarker levels differ between bipolar cases and controls, and genes regulate the expression of biomarkers in people with and without bipolar disorder in a similar way^20^. We could also posit that these findings might be specific to the Swedish population. Further studies are needed to affirm these assertions. Finally, it is possible that the slight deviations from normal distributions for biomarkers other than CRP could have resulted in false positive associations. However, the statistical tests used are robust to minor deviations in normality. False positives are a concern for all studies and may lead to variation in significant SNPs across studies, underscoring the importance of replication efforts.

The number of significant SNPs associated with disease-associated immune biomarkers differed depending on whether the sampling substrate was CSF or serum. GWS SNPs associated with blood serum biomarkers are more numerous than GWS SNPs in relation to CSF biomarkers. The reason might be the smaller sample size for CSF than blood serum. The associated SNPs and the genes where the SNPs localized are different between the same biomarker in CSF and serum. However, when comparing nominally associated SNPs from CSF and serum, effect sizes for YKL-40 and MCP-1 from CSF and serum are moderately correlated. Little correlation was observed in sCD14 while no correlation was found for TIMP-1 and TIMP-2. This mirrors findings from a previous study in this sample which observed correlations between CSF and serum levels for YKL-40 and MCP-1^20^ but not for TIMP-1 and TIMP-2. These convergent lines of evidence indicate that some biomarkers share genetic regulation across tissues while others do not.

There are limitations of this study to consider. First, given the hurdle of lumbar puncture to collect CSF, the sample size is limited and we were unable to identify a replication cohort. Despite this, we found numerous loci associated with immune biomarkers, but our sample size is almost certainly not sufficient to identify all relevant loci. Second, genotyping was conducted on different chips (PsychChip, Affymetrix 6.0, and Illumina OmniExpress) that provide incomplete overlap of directly genotyped markers. Although we use the imputed SNP dosages in order to increase the number of same SNPs shared by all three waves, the overlap is incomplete. Finally, there might be confounders associated with bipolar disorder such as smoking and alcohol use. Smoking and alcohol abuse might not only affect immune-related biomarker levels, but are also partly genetically mediated^60,61^. It is therefore possible that some significant genetic associations are due to the intermediary effect of smoking or alcohol consumption rather directly linked to biomarkers levels.

Despite the limitations, there are several strengths of this study. First, this is the first combined GWAS of peripheral and central immune biomarkers focusing on bipolar disorder to date, which shed light on the genetic regulation of the immune system in bipolar disorder. Second, we studied biomarkers measured in CSF that closely reflect the chemistry of the brain. Third, there is no evidence of heterogeneity for markers with the strongest association in our study, using Q-tests (*P* > 0.50) and *I*^2^ index (*I*^2^ = 0.00), while other SNPs showed high heterogeneity with Q-tests (*P* < 0.10) and *I*^2^ index (*I*^2^ > 10). We used the random-effects model for meta-analysis, which conservatively accounts for heterogeneity. We detected a number of SNPs associated with immune biomarker levels with large effect sizes. Compared to results from previous GWAS on bipolar disorder, the strength of the associations (i.e., effect sizes) between genetic variations and immune mediators is stronger than that with bipolar disorder itself^8,11,39^.

This study raises the possibility of using specific genetic markers as a proxy for immune biomarkers measured in CSF, which would be less cumbersome than collecting CSF through lumbar puncture. As sample size increases, the predictive ability will continue to improve. If replicated, the genetic markers may thus serve as indicators of biological processes implicated in psychiatric disorders. Indeed, specific genetic markers related to immune biomarkers profiles might be used to differentiate between different psychiatric diseases.

In summary, a number of biologically plausible SNPs significantly influencing immune biomarker levels in CSF and serum which demonstrated prior alterations in bipolar disorder have been identified in this study. Several of these SNPs are located in genes reported to be associated with bipolar disorder. The genetic variants associated with immune biomarker levels in CSF differ compared with those in serum. However, nominally significant variants showed correlated effect sizes for some biomarkers and this generally corresponded to whether the biomarker levels directly exhibited correlations between CSF and serum. The results of these GWAS can provide a route for the future investigations of genetic factors and immune biomarkers to aid in accurate diagnosis and development of treatments for bipolar disorder.

## Supplementary information

Supplement

## References

[1] Judd LL (2002). The long-term natural history of the weekly symptomatic status of bipolar I disorder. Arch. Gen. Psychiatry.

[2] Judd LL (2003). A prospective investigation of the natural history of the long-term weekly symptomatic status of bipolar II disorder. Arch. Gen. Psychiatry.

[3] World Health Organization. Mental disorders [Internet]. http://www.who.int/mediacentre/factsheets/fs396/en/ (2017).

[4] McGuffin P (2003). The heritability of bipolar affective disorder and the genetic relationship to unipolar depression. Arch. Gen. Psychiatry.

[5] Song J (2015). Bipolar disorder and its relation to major psychiatric disorders: a family-based study in the Swedish population. Bipolar Disord..

[6] Kieseppa T, Partonen T, Haukka J, Kaprio J, Lonnqvist J (2004). High concordance of bipolar I disorder in a nationwide sample of twins. Am. J. Psychiatry.

[7] Muhleisen TW (2014). Genome-wide association study reveals two new risk loci for bipolar disorder. Nat. Commun..

[8] Baum AE (2008). A genome-wide association study implicates diacylglycerol kinase eta (DGKH) and several other genes in the etiology of bipolar disorder. Mol. Psychiatry.

[9] Wellcome Trust Case Control Consortium. (2007). Genome-wide association study of 14,000 cases of seven common diseases and 3,000 shared controls. Nature.

[10] Soronen P (2010). Replication of GWAS of bipolar disorder: association of SNPs near CDH7 with bipolar disorder and visual processing. Mol. Psychiatry.

[11] Sklar P (2008). Whole-genome association study of bipolar disorder. Mol. Psychiatry.

[12] Rosenblat JD, McIntyre RS (2017). Bipolar disorder and immune dysfunction: epidemiological findings, proposed pathophysiology and clinical implications. Brain Sci..

[13] Muneer A (2016). Bipolar disorder: role of inflammation and the development of disease biomarkers. Psychiatry Investig..

[14] Cremaschi L (2017). Prevalences of autoimmune diseases in schizophrenia, bipolar I and II disorder, and controls. Psychiatry Res..

[15] Sellgren CM (2016). A genome-wide association study of kynurenic acid in cerebrospinal fluid: implications for psychosis and cognitive impairment in bipolar disorder. Mol. Psychiatry.

[16] Olsson SK, Sellgren C, Engberg G, Landén M, Erhardt S (2012). Cerebrospinal fluid kynurenic acid is associated with manic and psychotic features in patients with bipolar I disorder. Bipolar Disord..

[17] Bromander S (2012). Changes in serum and cerebrospinal fluid cytokines in response to non-neurological surgery: an observational study. J. Neuroinflammation.

[18] Maier B, Laurer HL, Rose S, Buurman WA, Marzi I (2005). Physiological levels of pro- and anti-inflammatory mediators in cerebrospinal fluid and plasma: a normative study. J. Neurotrauma.

[19] Sellgren CM (2019). Peripheral and central levels of kynurenic acid in bipolar disorder subjects and healthy controls. Transl. Psychiatry.

[20] Jakobsson J (2015). Monocyte and microglial activation in patients with mood-stabilized bipolar disorder. J. Psychiatry Neurosci..

[21] Isgren A (2015). Increased cerebrospinal fluid interleukin-8 in bipolar disorder patients associated with lithium and antipsychotic treatment. Brain Behav., Immun..

[22] Gottesman I, Gould T (2003). The endophenotype concept in psychiatry: etymology and strategic intentions. Am. J. Psychiatry.

[23] Sigitova E, Fisar Z, Hroudova J, Cikankova T, Raboch J (2016). Biological hypotheses and biomarkers of bipolar disorder. Psychiatry Clin Neurosci..

[24] Kauwe JSK (2014). Genome-Wide Association Study of CSF Levels of 59 Alzheimer’s Disease Candidate Proteins: Significant Associations with Proteins Involved in Amyloid Processing and Inflammation. PLoS Genet..

[25] Dehghan A (2011). Meta-analysis of genome-wide association studies in >80 000 subjects identifies multiple loci for C-reactive protein levels. Circulation.

[26] Kjaergaard AD, Johansen JS, Nordestgaard BG, Bojesen SE (2013). Genetic variants in CHI3L1 influencing YKL-40 levels: resequencing 900 individuals and genotyping 9000 individuals from the general population. J. Med. Genet..

[27] Ryden E (2009). A history of childhood attention-deficit hyperactivity disorder (ADHD) impacts clinical outcome in adult bipolar patients regardless of current ADHD. Acta Psychiatr. Scandinavica.

[28] Ryden E, Johansson C, Blennow K, Landen M (2009). Lower CSF HVA and 5-HIAA in bipolar disorder type 1 with a history of childhood ADHD. J. Neural Transm. (Vienna, Austria.: 1996).

[29] Jakobsson J (2013). Altered concentrations of amyloid precursor protein metabolites in the cerebrospinal fluid of patients with bipolar disorder. Neuropsychopharmacol.: Off. Publ. Am. Coll. Neuropsychopharmacol..

[30] Rolstad S (2015). Cognitive performance and cerebrospinal fluid biomarkers of neurodegeneration: a study of patients with bipolar disorder and healthy controls. PLoS ONE.

[31] Abecasis GR (2012). An integrated map of genetic variation from 1,092 human genomes. Nature.

[32] Song J (2016). Genome-wide association study identifies SESTD1 as a novel risk gene for lithium-responsive bipolar disorder. Mol. Psychiatry.

[33] Pe’er I, Yelensky R, Altshuler D, Daly MJ (2008). Estimation of the multiple testing burden for genomewide association studies of nearly all common variants. Genet. Epidemiol..

[34] Watanabe K, Taskesen E, van Bochoven A, Posthuma D (2017). Functional mapping and annotation of genetic associations with FUMA. Nat. Commun..

[35] de Leeuw CA, Mooij JM, Heskes T, Posthuma D (2015). MAGMA: generalized gene-set analysis of GWAS data. PLoS Comput. Biol..

[36] UCSC Genome Browser. UCSC Genome Browser on Human Feb. 2009 (GRCh37/hg19) Assembly [Internet]. http://genome-euro.ucsc.edu/cgi-bin/hgTracks?db=hg19&lastVirtModeType=default&lastVirtModeExtraState=&virtModeType=default&virtMode=&nonVirtPosition=&position=chr2%3A125382755%2D125383255&hgsid=222647055_OE4mND9W80rpAVkqZeEOh3xgK9 (2017).

[37] Lee JJ (2018). Gene discovery and polygenic prediction from a genome-wide association study of educational attainment in 1.1 million individuals. Nat. Genet..

[38] Yu H (2018). Five novel loci associated with antipsychotic treatment response in patients with schizophrenia: a genome-wide association study. Lancet Psychiatry.

[39] Djurovic S (2010). A genome-wide association study of bipolar disorder in Norwegian individuals, followed by replication in Icelandic sample. J. Affect. Disord..

[40] Akiyama M (2017). Genome-wide association study identifies 112 new loci for body mass index in the Japanese population. Nat. Genet..

[41] Feitosa MF (2018). Novel genetic associations for blood pressure identified via gene-alcohol interaction in up to 570K individuals across multiple ancestries. PLoS ONE.

[42] Nagel M (2018). Meta-analysis of genome-wide association studies for neuroticism in 449,484 individuals identifies novel genetic loci and pathways. Nat. Genet..

[43] Li Z (2017). Genome-wide association analysis identifies 30 new susceptibility loci for schizophrenia. Nat. Genet..

[44] Winkler TW (2015). The influence of age and sex on genetic associations with adult body size and shape: a large-scale genome-wide interaction study. PLOS Genet..

[45] Armstrong DL (2014). GWAS identifies novel SLE susceptibility genes and explains the association of the HLA region. Genes Immun..

[46] National Center for Biotechnology Information. Gene [Internet]. https://www.ncbi.nlm.nih.gov/gene/23314 (2017).

[47] Walker RM (2016). DNA methylation in a Scottish family multiply affected by bipolar disorder and major depressive disorder. Clin. Epigenetics.

[48] Zhao W (2016). A new bioinformatic insight into the associated proteins in psychiatric disorders. SpringerPlus.

[49] Bergen SE (2012). Genome-wide association study in a Swedish population yields support for greater CNV and MHC involvement in schizophrenia compared with bipolar disorder. Mol. Psychiatry.

[50] Etain B (2006). Genome-wide scan for genes involved in bipolar affective disorder in 70 European families ascertained through a bipolar type I early-onset proband: supportive evidence for linkage at 3p14. Mol. Psychiatry.

[51] Le-Niculescu H (2009). Convergent functional genomics of genome-wide association data for bipolar disorder: Comprehensive identification of candidate genes, pathways and mechanisms. Am. J. Med. Genet. Part B: Neuropsychiatr. Genet..

[52] Nurnberger JI (2014). Identification of pathways for bipolar disorder: a meta-analysis. JAMA Psychiatry.

[53] Maes HH, Neale MC, Eaves LJ (1997). Genetic and environmental factors in relative body weight and human adiposity. Behav. Genet..

[54] Allin KH, Bojesen SE, Nordestgaard BG (2009). Baseline C-reactive protein Is associated with incident cancer and survival in patients with cancer. J. Clin. Oncol..

[55] Dehghan A (2007). Genetic variation, C-reactive protein levels, and incidence of diabetes. Diabetes.

[56] Sesso HD (2003). C-reactive protein and the risk of developing hypertension. JAMA.

[57] Danesh J (2004). C-reactive protein and other circulating markers of inflammation in the prediction of coronary heart disease. N. Engl. J. Med..

[58] Kaptoge S (2010). C-reactive protein concentration and risk of coronary heart disease, stroke, and mortality: an individual participant meta-analysis. Lancet (Lond., Engl.).

[59] Naitza S (2012). A genome-wide association scan on the levels of markers of inflammation in sardinians reveals associations that underpin its complex regulation. PLoS Genet..

[60] Tobacco and Genetics Consortium. (2010). Genome-wide meta-analyses identify multiple loci associated with smoking behavior. Nat. Genet..

[61] Treutlein J, Rietschel M (2011). Genome-wide association studies of alcohol dependence and substance use disorders. Curr. Psychiatry Rep..

