# Supplementary material for: Genome-wide study of immune biomarkers in cerebrospinal fluid and serum from patients with bipolar disorder and controls
Source: Transl Psychiatry. 2020 Feb 5;10:58. doi: 10.1038/s41398-020-0737-6 (PMC7026056; doi:10.1038/s41398-020-0737-6)
Supplement: Supplementary file 1 — Supplement [file 41398_2020_737_MOESM1_ESM.docx]

Supplemental Material

Zhang, et al.

**List of abbreviations**

ADE Affective Disorder Evaluation

AUDIT Alcohol Use Disorders Identification Test

BMI Body mass index

CRP C-reactive protein

CSF Cerebrospinal fluid

DNA Deoxyribonucleic acid

DUDIT Drug Use Disorders Identification Test

GWAS Genome-wide association studies

GWS Genome-wide significant

IL-8 Interleukin 8

MCP-1 Monocyte chemoattractant protein-1

MDS Multidimensional scaling

M.I.N.I. Mini International Neuropsychiatric Interview

sCD14 Soluble cluster of differentiation

SNP Single nucleotide polymorphism

TIMP Tissue inhibitor of metalloproteinases

YKL-40 Chitinase-3-like protein-1


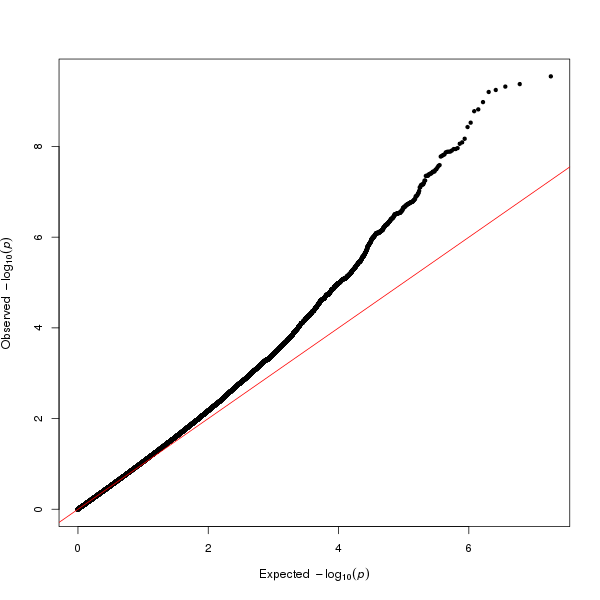


**YKL-40**


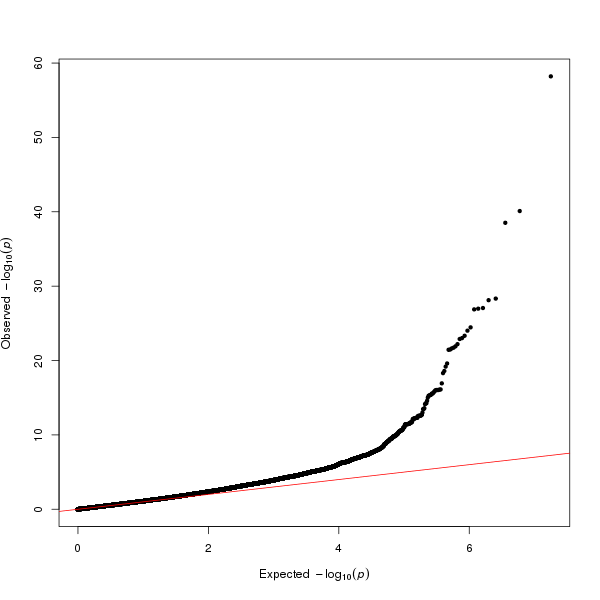


**MCP-1**


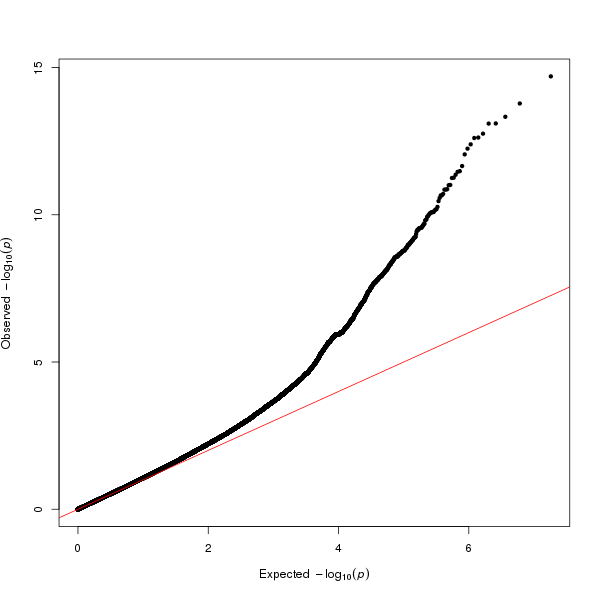


**sCD14**


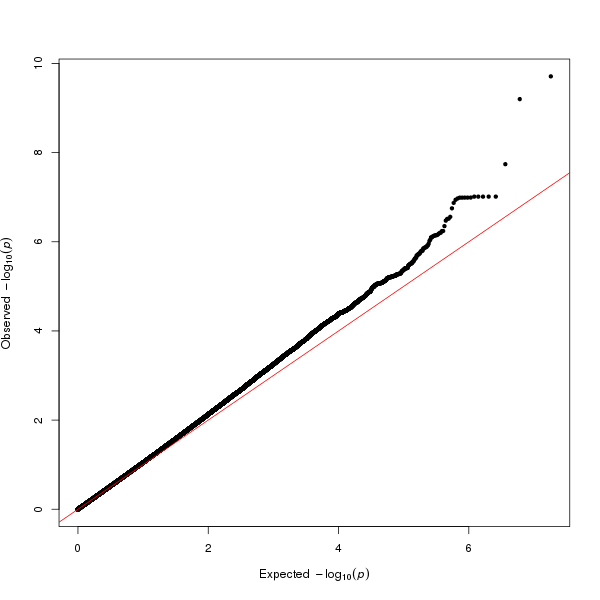


**TIMP-1**


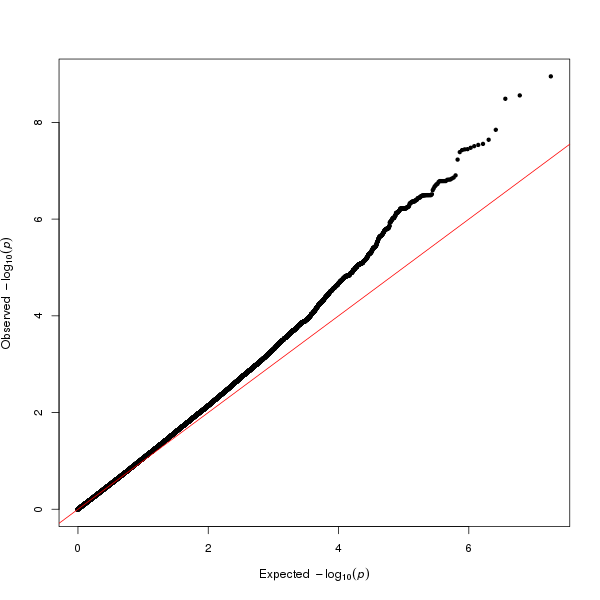


**TIMP-2**


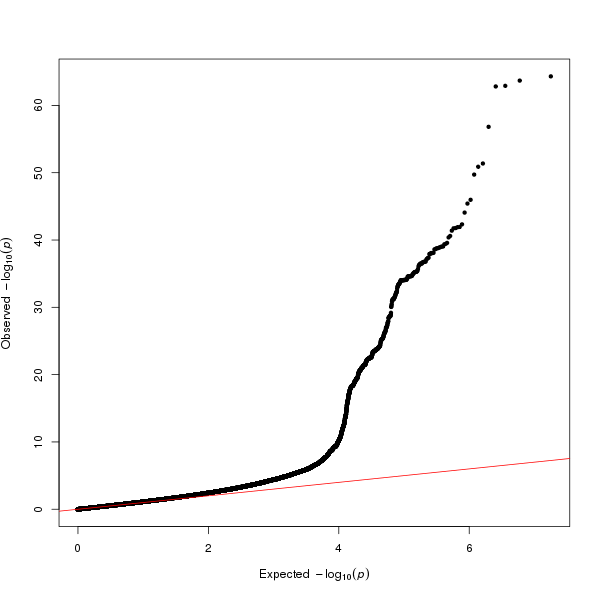


**IL-8**

**Figure S1. Quantile-quantile plots for six genome-wide scans of CSF immune biomarkers**

Quantile-quantile plots for each of the six biomarkers illustrate the relationship of the experimentally observed P-values (vertical axis) with the expected P-values of a null distribution (horizontal axis) for all SNPs that pass the standard quality control filters, have a minor allele frequency >1% and imputation info score >0.6. Each quantile-quantile plot involves around 5 600 000 SNPs.

λ=1.05

λ=1.09

λ=1.06

λ=1.06

λ=1.06

λ=1.11

**Figure S2. Expression of CSF immune biomarkers in 30 general tissue types (from GTEx v6)**


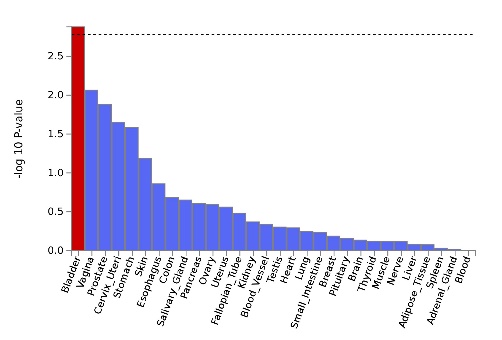

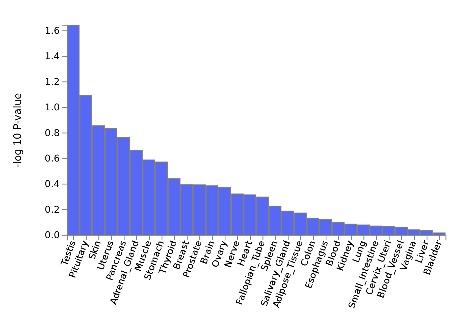

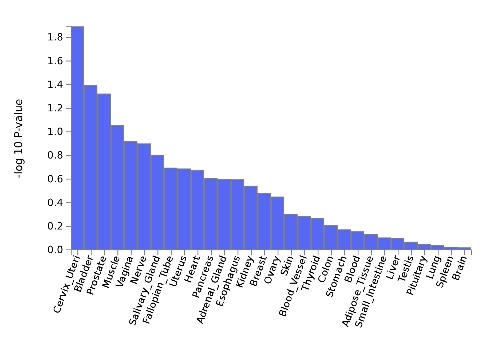

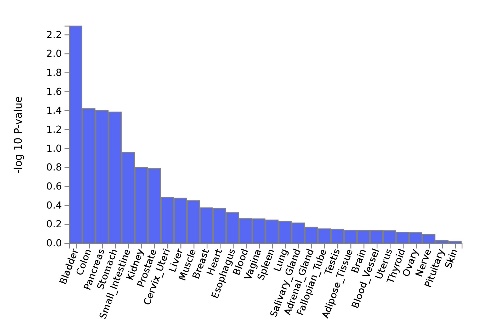

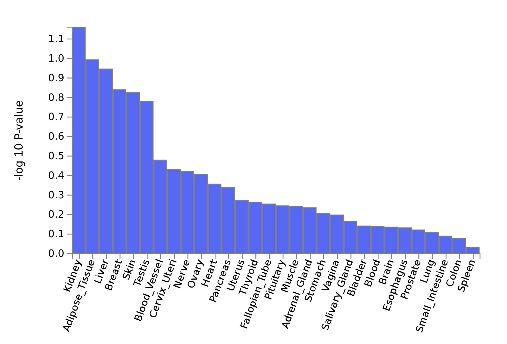

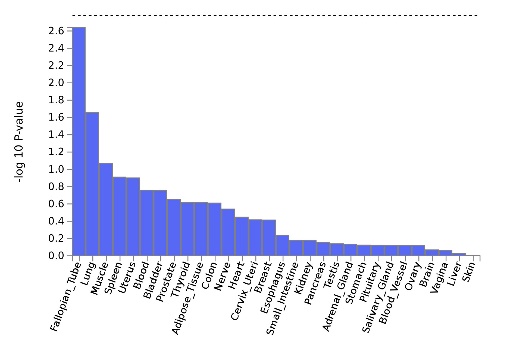


**YKL-40**

**MCP-1**

**sCD14**

**TIMP-1**

**TIMP-2**

**IL-8**


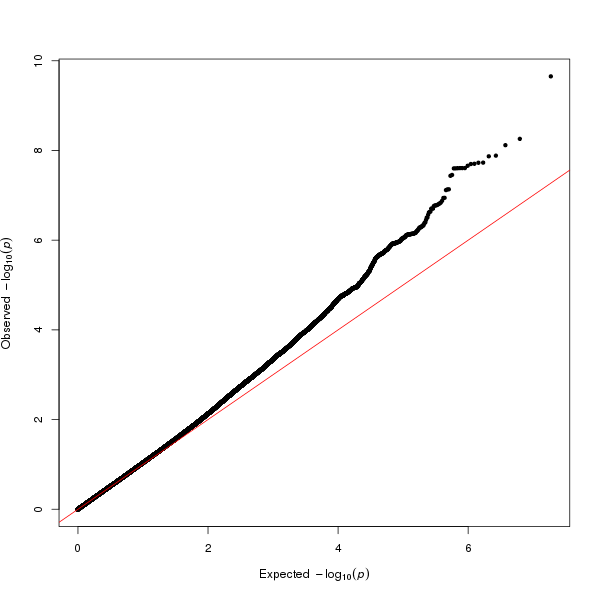

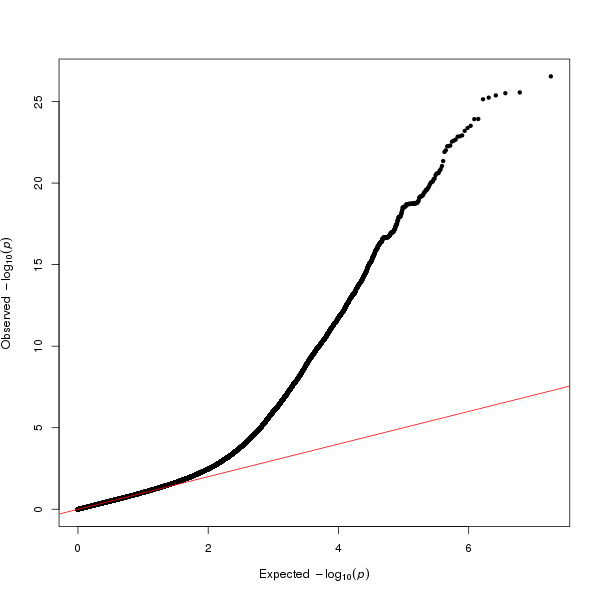


**YKL-40**


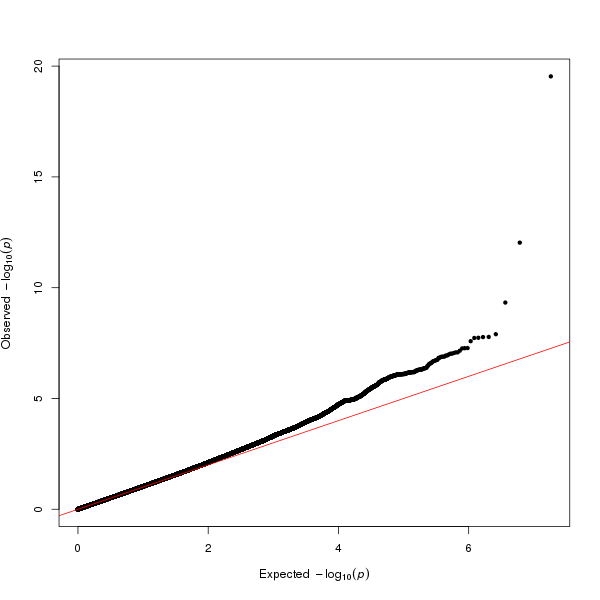


**MCP-1**


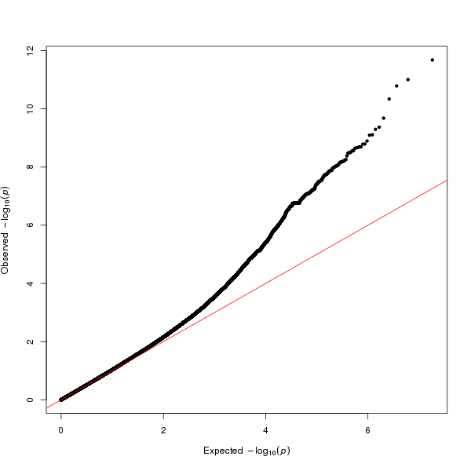


**sCD14**


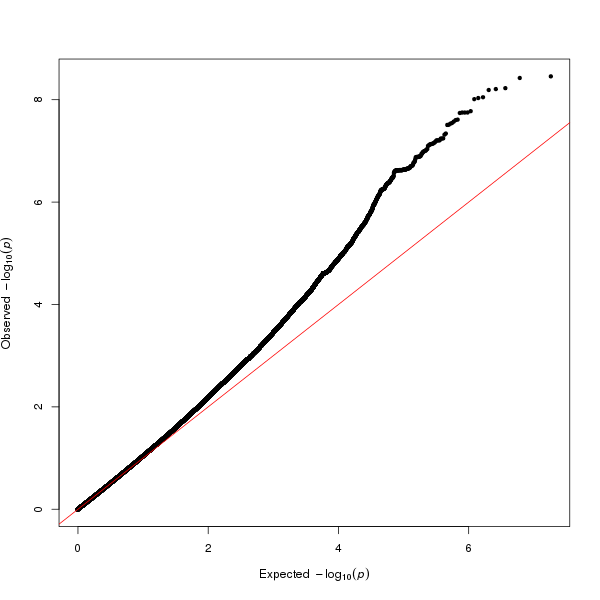


**TIMP-1**


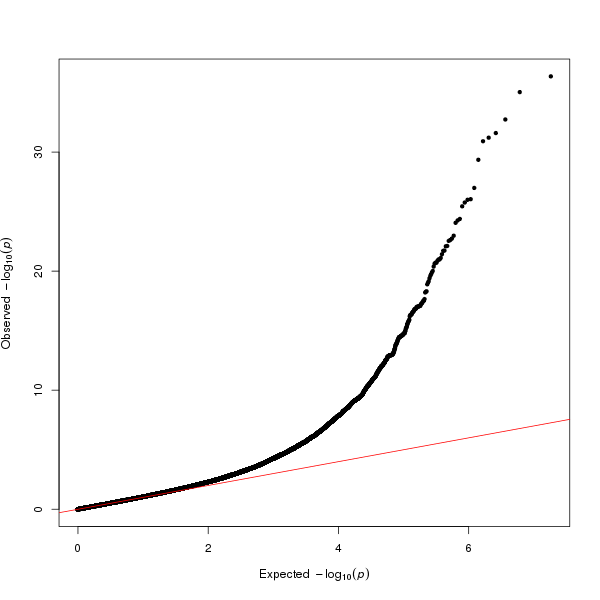


**TIMP-2**

**Log (CRP)**

**Figure S3. Quantile-quantile plots for six genome-wide scans of serum immune biomarkers**

Quantile-quantile plots show the relationship of the experimentally observed P-values (vertical axis) with the expected P-values of a null distribution (horizontal axis) for all SNPs that pass the standard quality control filters, have a minor allele frequency >1% and imputation info score >0.6. Each quantile-quantile plot involves around 5 340 000 SNPs.

λ=1.01

λ=1.04

λ=1.02

λ=1.02

λ=1.04

λ=1.05

**Figure S4. Expression of serum immune biomarkers in 30 general tissue types (from GTEx v6)**


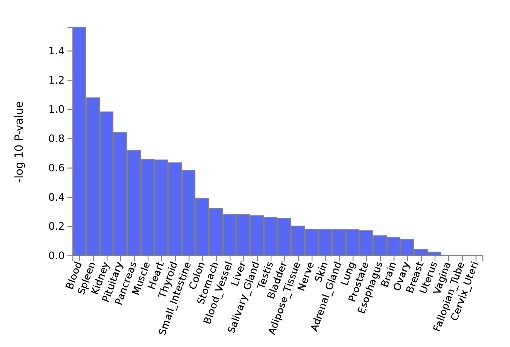

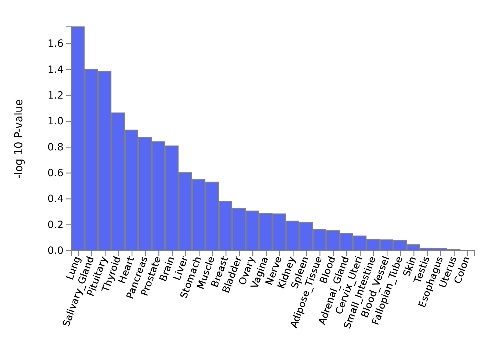

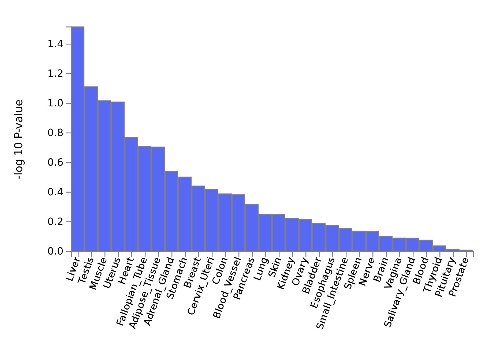

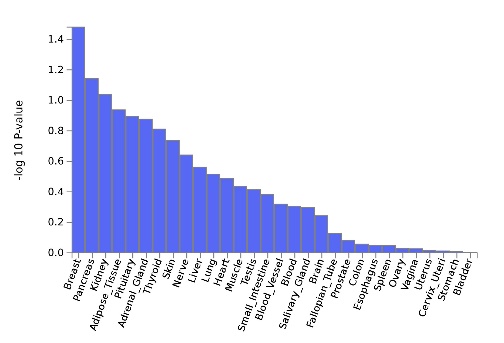

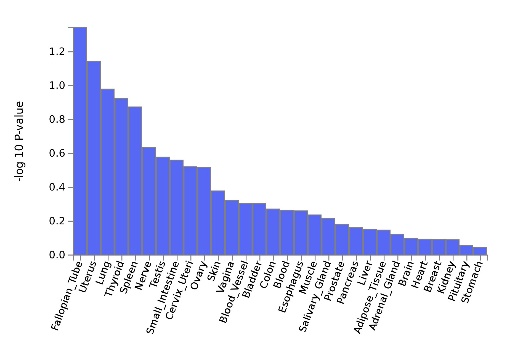


**YKL-40**

**MCP-1**

**sCD14**

**TIMP-1**

**TIMP-2**

**Log(CPR)**


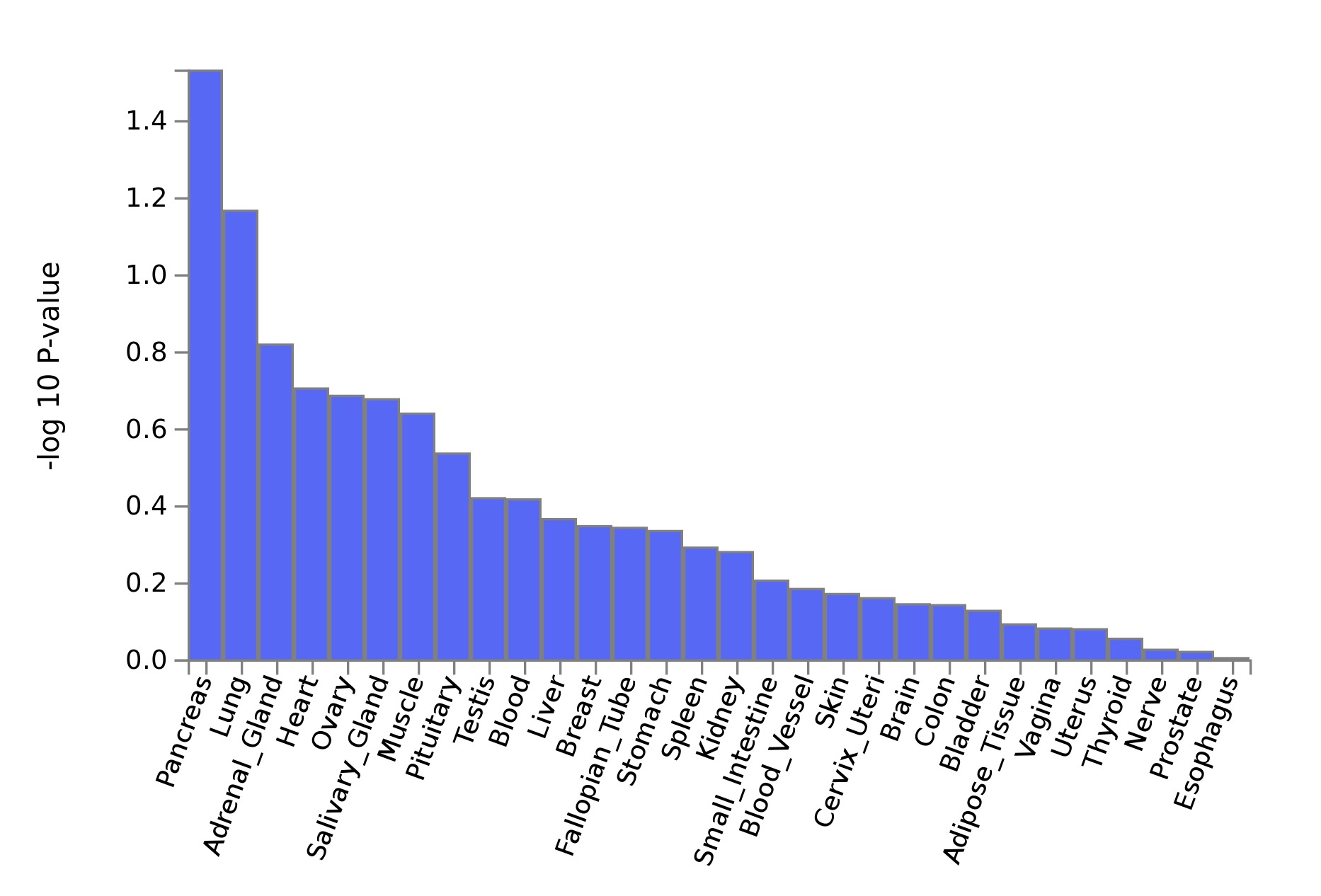


| **Table S1. Sample sizes by biomarker and wave** | | | | | |
| --- | --- | --- | --- | --- | --- |
|  |  | Wave 1 | Wave 2 | Wave 3 | Meta-analysis |
| CSF | Case:Control | 0:30 | 114:0 | 0:53 | 114:83 |
|  | TIMP-1 | 30 | 112 | 53 | 195 |
|  | TIMP-2 | 30 | 111 | 53 | 194 |
|  | sCD14 | 30 | 112 | 53 | 195 |
|  | YKL-40 | 30 | 106 | 53 | 189 |
|  | MCP-1 | 16 | 106 | 52 | 174 |
|  | IL-8 | 16 | 113 | 52 | 181 |
| Blood serum | Case:Control | 0:54 | 182:0 | 1:53 | 183:107 |
|  | TIMP-1 | 54 | 182 | 54 | 290 |
|  | TIMP-2 | 54 | 182 | 54 | 290 |
|  | sCD14 | 54 | 182 | 54 | 290 |
|  | YKL-40 | 54 | 182 | 54 | 290 |
|  | MCP-1 | 54 | 182 | 54 | 290 |
|  | CRP | 49 | 166 | 48 | 263 |
| *Abbreviations:* CSF=cerebrospinal fluid; TIMP=tissue inhibitor of | | | | | |
| metalloproteinases; sCD14=soluble cluster of differentiation 14; | | | | | |
| YKL-40=chitinase-3-like protein-1; MCP-1=monocyte chemoattractant | | | | | |
| protein-1; IL-8=interleukin 8; CRP=C-reactive protein. | | | | | |
| The numbers are subjects with available biomarker level information | | | | | |
| and passed genotyping quality control. | | | |  |  |

| **Table S2. Demographic and clinical characteristics of the study population** | | | | | | | | | |
| --- | --- | --- | --- | --- | --- | --- | --- | --- | --- |
|  | **CSF, no.(%) or median [IQR]** | |  | **Blood Serum, no.(%) or median [IQR]** | | | | |  |
| **Characteristic** | **Bipolar disorder (n=114)** | **Control (n=83)** | ***p* value** | **Bipolar disorder (n=184)** | | | **Control (n=107)** | | ***p* value** |
| **Sex,male** | 44(38.60) | 36(43.37) | 0.60**^1^** | 67(36.4) | | | 46(43.0) | | 0.32**^1^** |
| **Age,yr** | 37[30-51] | 36[27.5-47] | 0.29**^2^** | 36.5[30.0-48.0] | | | 35.0[27.5-44.0] | | 0.18**^2^** |
| **BMI** | 24.8[22.1-27.8] | 23.3[21.6-25.7] | **0.018^2^** | 25.03[22.18-27.75] | | | 23.31[21.65-25.58] | | **0.003^2^** |
| **TIMP-1*(ng/mL)** | 34.6**^a^**[29.5-39.7] | 32.0[28.3-32.8] | **0.038^2^** | 203.6**^f^**[172.5-231.9] | | | 191.6[169.3-218.7] | | **0.027^2^** |
| **TIMP-2*(ng/mL)** | 65.9**^b^**[56.5-78.6] | 60.9[51.5-74.3] | 0.090**^2^** | 63.8**^f^**[56.7-79.2] | | | 64.5[57.6-74.2] | | 0.72**^2^** |
| **sCD14*(ng/mL)** | 52.8**^a^**[34.0-69.7] | 43.4[28.9-55.1] | **0.021^2^** | 1457.5**^f^**[1311.3-1675.1] | | | 1360.7[1234.1-1541.4] | | **0.002^2^** |
| **YKL-40*(ng/mL)** | 81.6**^c^**[55.1-109.4] | 61.7[49.2-90.6] | **0.025^2^** | 24.1^f^[18.8-32.2] | | | 20.8[15.8-26.9] | | **<0.001^2^** |
| **MCP-1*(pg/mL)** | 491.0**^d^**[418.0-601.0] | 440.5**^e^**[379.2-522.2] | **0.004^2^** | 592.0**^f^**[489.0-715.5] | | | 561.0[455.5-687.0] | | 0.074^2^ |
| **IL-8*(pg/mL)** | 33.1^f^[27.6-39.7] | 27.9^e^[24.4-35.4] | **<0.001^2^** | - | | | - | | - |
| **CRP*(mg/L)** | - | - | - | 0.8**^g^**[0.2-2.5] | | | 0.7**^h^**[0.4-1.8] | | 0.98**^2^** |
| **Log*(CRP)** | - | - | - | -0.2**^g^**[-1.4-0.9] | | | -0.3**^h^**[-1.0-0.6] | | 0.98**^2^** |
| Bold *p* values are significant at the 0.05 level | | | | | | | | |  |
| **^1^** Analyzed by Fisher's exact test. | | | | | | | | |  |
| **^2^** Analyzed by Mann-Whitney *U* test. | | | | | | | |  |  |
| *Missing data: **^a^**N=2, **^b^**N=3 , **^c^**N=8, **^d^**N=8, **^e^**N=15, ^f^N=1, **^g^**N=17, **^h^**N=11 | | | | |  |  | |  |  |

| Table S3. MAGMA Gene-Set Analysis for biomarkers with GWS SNPs | | | | | | | |
| --- | --- | --- | --- | --- | --- | --- | --- |
| Biomarker | **Gene Set** | **N genes** | **Beta** | **Beta STD** | **SE** | **P** | **P_bon_** |
| sCD14_serum | Curated_gene_sets:zwang_class_1_transiently_induced_by_egf | 458 | 0.175 | 0.027 | 0.037 | 1.0035e-06 | **0.011*** |
|  | GO_mf:go_apolipoprotein_binding | 15 | 0.919 | 0.026 | 0.208 | 5.2139e-06 | 0.056 |
|  | GO_bp:go_negative_chemotaxis | 37 | 0.516 | 0.023 | 0.131 | 4.1377e-05 | 0.442 |
|  | GO_bp:go_epiboly | 20 | 0.623 | 0.021 | 0.159 | 4.439e-05 | 0.474 |
|  | Curated_gene_sets:kegg_proximal_tubule_bicarbonate_reclamation | 22 | 0.580 | 0.020 | 0.154 | 8.6334e-05 | 0.921 |
|  | Curated_gene_sets:pid_ecadherin_stabilization_pathway | 42 | 0.432 | 0.021 | 0.116 | 9.2164e-05 | 0.983 |
| YKL40_serum | GO_bp:go_regulation_of_transcription_involved_in_cell_fate_commitment | 19 | 0.861 | 0.028 | 0.206 | 1.5003e-05 | 0.160 |
|  | GO_mf:go_protein_kinase_c_binding | 50 | 0.393 | 0.021 | 0.102 | 5.9141e-05 | 0.631 |
|  | GO_mf:go_protein_complex_scaffold | 66 | 0.329 | 0.020 | 0.088 | 9.0723e-05 | 0.968 |
| Log(CPR)_serum | GO_mf:go_s100_protein_binding | 12 | 0.977 | 0.025 | 0.250 | 4.5929e-05 | 0.490 |
|  | GO_bp:go_regulation_of_voltage_gated_calcium_channel_activity | 22 | 0.689 | 0.024 | 0.182 | 7.9533e-05 | 0.849 |
| TIMP1_serum | GO_bp:go_coenzyme_a_biosynthetic_process | 11 | 0.853 | 0.021 | 0.222 | 5.9567e-05 | 0.636 |
| sCD14_csf | Curated_gene_sets:biocarta_gh_pathway | 28 | 0.597 | 0.023 | 0.156 | 6.3259e-05 | 0.675 |
| IL8_csf | Curated_gene_sets:elvidge_hif1a_targets_up | 61 | 0.381 | 0.022 | 0.100 | 6.7781e-05 | 0.723 |
|  | Curated_gene_sets:reactome_generic_transcription_pathway | 328 | 0.177 | 0.024 | 0.047 | 8.9076e-05 | 0.951 |
| P_bon_ is the p value after Bonferroni correction.  *significant gene sets after Bonferroni correction.  The table displays gene sets with P_bon_ < 1. | | | | | | | |
